# Severe recurrent pneumonia in children: Underlying causes and clinical profile in Vietnam

**DOI:** 10.1016/j.amsu.2021.102476

**Published:** 2021-06-09

**Authors:** Kim Lam Hoang, Anh Tuan Ta, Van Thang Pham

**Affiliations:** a Department of Pediatrics, Hanoi Medical University, Ton That Tung, Hanoi, Viet Nam; b Vietnam National Children's Hospital, La Thanh, Hanoi, Viet Nam

**Keywords:** Severe pneumonia, Recurrent pneumonia, Underlying causes, PICU, Vietnam

## Abstract

**Background:**

There is still limited data on severe recurrent pneumonia in children, especially in developing countries as Vietnam. This study was conducted to identify the underlying causes and clinical profile of children with severe recurrent pneumonia admitted to the Pediatric Intensive Care Unit (PICU), National Children's Hospital.

**Methods:**

This was a prospective and descriptive study on 110 children with severe pneumonia admitted to the PICU from November 2019 to August 2020. Data were collected to investigate the clinical profile and underlying diseases.

**Results:**

Severe recurrent pneumonia accounted for 29.4%. Underlying causes were diagnosed in 91.8% of sRP children, in which the most common causes were abnormalities in respiratory, cardiovascular system and immune disorders. 74.5% of sRP children admitted to ICU had been previously intubated or ventilated, 34.5% had shock, 7.3% had multiple organ failure. Recurrent lesions on chest x-ray in the same lobe accounted for 18.2%.

**Conclusions:**

The majority of patients with severe recurrent pneumonia had an underlying disease. Comprehensive management is necessary for severe recurrent pneumonia.

## Highlights

• Highlight:
• Severe recurrent pneumonia accounted for 29.4% of pneumonia cases and 14.4% of all cases admitted to PICU.
• Underlying causes were diagnosed in 91.8% of severe recurrent pneumonia children.
• In Vietnam, most common causes of sRP were abnormalities in respiratory, cardiovascular system and immune disorders.

## 1 Introduction

Pneumonia is the most common disease and also the leading cause of death in children worldwide [1]. It is estimated that about 6% of young children will experience at least one episode of pneumonia in the first 2 years of life [2,3]. In low-and-middle-income countries, the rate of pneumonia is still high and pneumonia remains being the major cause of morbidity and mortality of young children [4]. Although there has had general improvement in living status, nutrition and vaccination, more than 700,000 children less than 5 years died from pneumonia globally [5].

Recurrent pneumonia (RP) is defined as at least 2 pneumonia episodes in a 1-year period or at least 3 episodes at any time, without any clinical symptoms and chest X-ray lesions between pneumonia episodes [6,7]. It is up to 9% of children with pneumonia will progress to RP, even in developed countries [8]. In which, more than 80% of children have an underlying illness [6,[9], [10], [11]]. Among RP children, the group of children with severe pneumonia admitted to the Pediatric Intensive Care Unit (PICU) has a high morbidity and mortality rate [12]. However, to the best of our knowledge, the data on severe recurrent pneumonia (sRP) admitted to PICU is still insufficient, especially in developing countries such as Vietnam. Thus, this study was conducted to identify the underlying causes and clinical profile of children with sRP admitted to the Pediatric Intensive Care Unit.

## 2 Materials and methods

Registration and ethics: Research Registry number is stated, in accordance with the declaration of Helsinki. Unique identifying number: researchregistry6750.

https://www.researchregistry.com/browse-the-registry#home/registrationdetails/6079bbcee0b507001bdfc275/

This was a prospective and descriptive cohort study. All patients classified as severe recurrent pneumonia, admitted to Pediatric Intensive Care Unit, National Children's Hospital from November 2019 to August 2020 were recruited. Diagnosis of pneumonia was based on cough, chest wall in-drawing and/or difficult breathing and tachypnea, fever and lobar or bronchopneumonic infiltration demonstrated by chest X-ray. Patients were diagnosed as severe pneumonia if pneumonia with one major criterion or two minor criteria according to the Pediatric Infectious Diseases Society (PIDS) and the Infectious Diseases Society of America (IDSA) [13,14]. Recurrent pneumonia was defined as two episodes of pneumonia occurring in 1 year or three episodes of pneumonia occurring in any time frame; without any clinical symptoms or chest X-ray lesions between pneumonia episodes [7]. This study has been reported in line with the STROCSS criteria [15].

### Identifying the underlying causes

All sRP children admitted to PICU were investigated previous medical history, medical history, physical examination and underwent initial tests to diagnose the causes. Information was collected in the medical record forms for sRP patients. Patients were divided into groups based on having recurrent lesion in the same lobe or new lesion in different lobe before undergoing several investigational tests to identify the underlying causes. Pulmonary parenchymal or airway abnormalities were confirmed by chest CT scan and/or bronchoscopy, bronchoalveolar lavage tests or lung biopsy. On the other hand, cardiovascular abnormalities were associated with echocardiography, computed tomography, or cardiac catheterization. Immune disorders were determined by quantitative immunoglobulins (IgG, IgA, IgM, IgE), lymphocyte counting test of T-CD3, T-CD4, T-CD8 using flow cytometry, or specific disease criteria (acute leukemia, hemophagocytic lymphohistiocytosis, etc.). While the aspiration syndrome was clinically diagnosed, genetic diseases were investigated by genetic analysis. Finally, transient recurrent wheezing was diagnosed in children under 3 years of age, with three episodes of wheezing, after the episodes the child has no symptom with normal physical and mental development, no specific underlying cause, without risk of API classification (Asthma Predictive Index) [16].

The project was approved by the Hanoi Medical University Institutional Ethical Review Board. IRB-VN01.001/IRB00003121/FWA 00004148 and the Vietnam National Children's Hospital. All participants' parents or supervisors have given informed written consent.

## 3 Results

There were 771 patients admitted to Pediatric Intensive Care Unit (PICU) during a 10-month span. In which, there were 374 (48.5%) with pneumonia and 110 (14.3%) met the criteria for sRP. Demographic characteristics of children with sRP were presented in Table 1. The average age of sRP diagnosis was 15.0 months, whereas age at the first episode of pneumonia was 11.6 months. Among children with sRP, the majority was male, being twice compared with female. Most patients with sRP came from rural regions of Vietnam, being 3.4 times compared with ones from urban regions.

**Table 1:** Demographic features of sRP patients.

Characteristics	Value
Age related to diagnosis	
Age of admission (month)	20.8 ± 24.5
Age of the 1st episode of pneumonia (month)	11.6 ± 29.2
Age of RP (month)	15.0 ± 30.4
Gender	
Male/Female	75 (68.2%)/35 (31.8%)
M/F ratio	2.14/1
*P*-value	<0.001
Type of dwelling	
Urban	25 (22.7%)
Rural	85 (77.3%)

Clinical features and chest X-ray lesions were indicated in Table 2. The most common symptom was cough, which occurred in 99.1% patients. Other common symptoms in children with sRP included lung crackles or wheezes, fever and wheezing. The majority of children with sRP admitted to PICU were previously intubated or ventilated (74.5%), 34.5% with circulatory failure, and 7.3% with multi-organ failure. Respiratory failure with increased PaCO2 was reported in 63.6% of sRP children. Local and repeated chest X-ray lesions in one lung lobe accounted for 18.2%.

**Table 2:** Clinical characteristics and chest X-ray lesions of sRP.

Clinical symtoms				n	%
1. Fever				66	60.0
2. Cough				109	99.1
3. Wheezing				69	62.7
4. Lung auscultation	⁃Crackles			95	86.4
	⁃Wheezes			67	60.9
	⁃No rales			14	12.7
5. Abnormal heart sounds (murmur, galop)				13	11.8
6. Signs of heart failure				14	12.7
Severe conditions on admission to ICU				**n**	**%**
1. Respiratory support prior to admission to ICU					
⁃Oxygen via face masks or nasal prongs				26	23.6
⁃Non-invasive ventilation (N-PPV or CPAP)				2	1.8
⁃Intubated and mechanical ventilated				82	74.5
2. Respiratoty support 24 h after admission to ICU					
⁃Oxygen via face masks or nasal prongs				4	3.6
⁃Non-invasive ventilation (N-PPV or CPAP)				4	3.6
⁃Regular ventilation				88	80.0
⁃High-frequency oscillatory ventilation (HFOV)				14	12.7
3. Type of respiratory failure		⁃Decrease of PaO_2_		110	100.0
		⁃Increase of PaCO_2_		70	63.6
4. Oxygenation Index (OI) 24 h after admission to ICU		⁃OI < 4		33	30.0
		⁃4 ≤ OI < 8		36	32.7
		⁃8 ≤ OI < 16		18	16.4
		⁃OI ≥ 16		17	15.5
5. Shock				38	34.5
6. Vasoactive-Inotropic Score (VIS) 24 h after admission to ICU			⁃VIS < 10	1	0.9
			⁃10 ≤ VIS < 25	26	23.6
			⁃VIS ≥ 25	11	10.0
7. Multiple organ dysfuntion syndrome				8	7.3
Chest X-rays				**n**	**%**
1. Recurrent lesions in the same lobes				20	18.2
2. Lesions in different/multiple lobes				90	81.8

Underlying causes of sRP were represented in Table 3. The leading causes of sRP was respiratory abnormalities, accounted for 28.2%; the second common causes of sRP were immune disorders; and the following causes was congenital heart diseases and aspiration syndrome. There were still 8.2% patients with normal previous medical history being admitted to PICU for sRP treatment.

**Table 3:** Underlying causes of sRP patients.

Underlying causes	n	%
Congenital heart diseases	17	15.5%
Immune dissorders	24	21.8%
Respiratory abnormalities	31	28.2%
Pulmonary hemorrhagic syndrome	2	1.8%
Post-infectious bronchiolitis obliterans (PIBO)	5	4.5%
Aspiration syndrome	12	10.9%
Neuromuscular disorders	6	5.5%
Recurrent wheezing	4	3.6%
Unknown	9	8.2%

## 4 Discussion

Severe recurrent pneumonia accounted for 29.4% of pneumonia cases and 14.4% of all cases admitted to PICU, which was much higher than the rate of RP in previous research (7.7–9.0%) [6,10,11,17,18]. As a result. sRP become a serious situation in many developing countries, including Vietnam. In our study, 74.5% of sRP children admitted to PICU had been previously intubated or ventilated, 34.5% had shock, and 7.3% had multiple organ failure. Therefore, this group of children with severe pneumonia had a very high morbidity and mortality rate [19]. The average age of sRP was 20.8 months in our hospital, being similar to the data reported by Ciftci et al. (23.6 months) [10]. Most patients came from rural area, giving a fact that the limitation of medical facility as well as the lower awareness of parents living in low-income regions may result in a severe condition of their children [4,20].

In our study, most cases of sRP were found to be associated with an underlying cause after running several tests and examination. An important and helpful method to reveal the underlying conditions in many cases with sRP was investigating previous medical history. sRP children with a history of psychomotor retardation may indicate underlying neuropathy or inhalation syndrome. History of psychomotor retardation or malnutrition may be associated with respiratory or cardiovascular abnormalities. A history of recurrent wheezing may suggest groups of respiratory diseases (respiratory abnormalities, PIBO, recurrent wheezing), inhalation syndrome, or immune disorders. A history of meal-related coughing or choking was related to inhalation syndrome, or neuropathy. And a history of severe or persistent/recurrent infection in other organ other than the lung, or a history of long-term corticosteroid therapy may indicate an underlying cause of immune disorder. After collecting the clues for the causal status, many tests including chest CT scan, bronchoscopy, ECG, ultrasound, or blood tests may be performed to confirm the diagnosis. Nevertheless, there were still 8.2% of cases with unknown causes among sRP children in our results. Other studies also reported about 3–14% of sRP cases cannot identify the causes after ruling out all possible explanations [6,[9], [10], [11]]. Therefore, it is needed to follow-up closely these children to find out whether the causes may be revealed afterwards.

Mohammad et al. found that the leading cause of RP was aspiration syndrome (51.75%), then recurrent wheezing (20.17%) and congenital heart disease (20.17%) [9]. In contrast, Hossain reported that pulmonary tuberculosis played the most important role in RP causes, accounting for 23.3%, then congenital heart disease (16.6%), cystic fibrosis (13.3%), immune deficiency (10%), and bronchial asthma (10%) [21]. In our study, the most frequent underlying illness associated with sRP was respiratory abnormalities (28.2%), followed by immune disorders (21.8%), congenital heart diseases (15.5%) and aspiration syndrome (10.9%). Patients’ respiratory abnormalities were a diverse group with different disorders in structures of airway tract, thorax, and pulmonary parenchyma [8]. Patients with normal respiratory system may recover from pneumonia quickly and have less chance of developing severe condition. On the other hand, children born with abnormalities in respiratory system may be at higher risk of progression to sRP. These deformities led to the requirement of non-invasive or invasive ventilation, thus the rate of PICU admission was higher.

Many immune disorders may be found in a patient with sRP, such as low T-CD4 lymphocyte, deficiency in function and quantity of neutrophils, and even HIV. Children with immune disorders are at high risk of sRP. This is a result of the infection of unusual organisms or involvement of multiple sites of infection in addition to the lungs [21,22]. Congenital heart disease is also an important cause of sRP in children. Congenital acyanotic heart diseases with left-to-right shunts increase the susceptibility to respiratory infection, thus also raise the rate of RP and sRP [23]. Children with heart diseases also need a comprehensive management, including respiratory ventilation, antibiotics and also heart disease support. Consequently, the rate of PICU hospitalization is higher than other children.

Aspiration syndrome accounted for 10.9% of the underlying causes in our patients. Aspiration syndrome may be associated with gastro-esophageal reflux disease, pharyngeal incoordination, and foreign body aspiration [21]. The diagnosis of aspiration syndrome is a challenge for clinicians since it bases upon the clinical examination and medical history report. Patients also are often admitted in severe conditions thus it prevents doctors from digging deeply into the underlying cause of sRP.

Most children in our study were admitted to PICU in urgent condition, which may limit the investigation of underlying causes. Besides, clinical guidelines for causal diagnosis in severe recurrent pneumonia has still not been available yet, making it difficult for physicians to practice in patients with sRP. Thus, some tests had to be delayed until patients recovered and a huge range of tests had to be run to cover all the possible causes in those patients without evidences of appropriate timing or optimal sequence of investigations. Moreover, this study mainly found the associated conditions in sRP patients, while the effect of these conditions to pneumonia episodes, including severity, PICU admission, intubation duration, as well as recurrent pneumonia prediction had not been established.

In this study, most patients with sRP were related to underlying causes, which may be the trigger of this episode or needed to handle simultaneously to improve patients' status. Future studies should focus on the difference in diagnosis and treatment in each group with specific underlying cause, as well as the impact of each condition in patients’ management and prevention.

## 5 Conclusions

Severe recurrent pneumonia accounts for a major part in clinical scenario in pediatric intensive care unit. More than 90% of patients with severe recurrent pneumonia had an underlying disease, in which, the common causes are abnormalities in respiratory system, immune disorders, congenital heart diseases, and aspiration syndrome. A comprehensive management is necessary for severe recurrent pneumonia since the mortality rate is high and multi-organ failure may occur in these patients.

## Author disclosure statement

Nothing to disclose.

## Funding statement

No funding sources.

## Provenance and peer review

Not commissioned, externally peer-reviewed.

## Please state any conflicts of interest

None.

## Please state any sources of funding for your research

None.

## Ethical approval

The project was approved by the Hanoi Medical University Institutional Ethical Review Board. IRB-VN01.001/IRB00003121/FWA 00004148 and the Vietnam National Children's Hospital.

## Consent

The publication of this study has been consented by the relevant patient. Informed consent was obtained from the parents for the report publication.

## Author contribution

Kim Lam Hoang and Anh Tuan Ta: Contributed equally to this work; Designed the study, did the data collection, the data analysis, the writing paper.

Van Thang Pham: Revised the manuscript.

All authors have read and approved the final version of the manuscript.

## Registration of research studies

1. Name of the registry: Research Registry
2. Unique Identifying number or registration ID: researchregistry6750
3. Hyperlink to your specific registration (must be publicly accessible and will be checked):https://www.researchregistry.com/browse-the-registry#home/registrationdetails/6079bbcee0b507001bdfc275/

## Guarantor

Dr. Kim Lam Hoang, M.D.

Email address: hoangkimlam@hmu.edu.vn.
